# Use of modern contraceptives on the basis of disability severity in Uganda: analysis of the 2016 demographic and health survey

**DOI:** 10.3389/frph.2025.1611713

**Published:** 2025-08-07

**Authors:** Tara Casebolt, Molly Hardiman, Taylor Diaz-Leon Guerrero

**Affiliations:** ^1^ Department of Public Health Sciences, Parkinson School of Health Sciences and Public Health, Loyola University Chicago, Chicago, IL, United States; ^2^ Morrissey College of Arts and Sciences, Boston College, Chestnut Hill, MA, United States

**Keywords:** disability, contraception, Uganda, birth control, accessibility, family planning

## Abstract

**Background:**

Women with disabilities have faced difficulties with accessing family planning services for decades and in many different settings. These women have both been denied access to contraception because of an inaccurate belief of the asexuality of people with disabilities and been forced or coerced into using contraceptives because of the belief they should not have children.

**Objective:**

This study uses Demographic and Health Survey data to analyze the association between disability and modern contraceptive use in Uganda. A disability severity indicator was used based upon recommendations from the Washington Group on Disability Statistics.

**Methods:**

Bivariate analysis was performed using chi-squares to determine if a significant association exists between modern contraceptive use and disability. Logistic regressions were run to generate odds ratios for crude adjusted models which took demographic data and covariates into consideration.

**Results:**

Disability was not found to be significantly associated with modern contraceptive use. Women with disabilities did not have significantly different odds of using a modern form of contraceptive when compared to women without disabilities in both the crude and adjusted models.

**Conclusions:**

These findings are inconsistent with previous studies conducted in Uganda. Additional research should be conducted to determine if there are disparities in the type of contraceptive used, sustainability of the method, and whether there is an unmet need for contraceptives. Based on the findings of this study, it is clear that women with disabilities use contraceptives. Therefore, it is essential for family planning services to be accessible to women regardless of functional limitations.

## 1 Introduction

Approximately 1.3 billion people, or 16% of the global population, experience a significant disability (1). Though progress has been made in addressing health inequities faced by persons with disabilities, there is still a lack of resources available that would allow for persons with disabilities to obtain the highest attainable standard of health, an established human right (1). Access to sexual and reproductive health services is essential for all women to obtain a proper standard of health, however, women with disabilities face unique barriers to care when accessing these services that hinder their ability to properly experience them. Research remains scarce regarding the difficulties women with disabilities experienced regarding accessing care which not only affects health seeking behaviors, but the quality of care experienced. The lack of data is partially due to the fact that sexual and reproductive health services and disability data are not commonly collected within the same dataset. As a population that is particularly at risk of health and socioeconomic vulnerabilities, it is important to assess the role of disability in accessing contraception and other sexual and reproductive health services together. The lack of research exemplifies the demand to view persons with disabilities with individual autonomy who have rights to reproductive health and quality care (2, 3).

According to the 2016 Uganda Demographic and Health Survey (UDHS), 35% of currently married women 15–49 years of age reported use of modern conception, increasing from 14% in 2000–01. Injectables are the most commonly used modern method among sexually active currently married and unmarried women, with 19% and 21% reporting using this method respectively. Implants are the second most common method among married women with 6% reporting use, while male condoms are the second most common method for unmarried women at 14%. Prevalence of modern contraceptive use was higher among unmarried women, women with more than a secondary education, wealthier women, and women living in rural areas (4).

A few qualitative studies have been conducted in Uganda regarding disability and reproductive health that revealed some commonly reported barriers women with disabilities experience in accessing these services. Inhibited access is often due to physical inaccessibility, poor treatment from health care workers, social discrimination, and high costs of service. Physical inaccessibility involves the inability to access services because of unfriendly physical facilities, or inaccessible infrastructure due to lack of ramps, personnel for assistance, wheelchairs, disability-friendly beds, and other such needs. Additionally, women with disabilities face the challenges of long distances to health care facilities and long queues at facilities when they may not have the capabilities to travel and wait for long periods of time. Negative attitudes from health care providers and societal conceptions of women with disabilities also prevent access to sexual and reproductive health services and reduce the quality of care received. Many providers and members of society in general have the notion that women with disabilities are not or should not be sexually active, and thus do not need contraception or other sexual and reproductive health services (5). Lastly, as persons with disabilities are more likely to live in poverty, high costs of services contribute to making sexual and reproductive health services inaccessible to them (6). A higher proportion of persons with disabilities, compared to those without disabilities, live under national or international poverty lines, therefore, financially, they are less likely to be able to pay for such services (1). In many countries, basic contraceptive services are supposed to be provided free of charge at government managed healthcare facilities. However, many women report being charged for these services. Because of the high poverty rates among people with disabilities, this leaves women with disabilities in challenging financial position where they must decide between receiving essential health services or using their limited funds for other needs (5).

Some studies in Uganda have addressed accessibility of sexual and reproductive health services and contraception. However, these tend to focus on particular groups such as only women with physical disabilities or women living in urban areas. Limiting studies to specific subsets of the disabled population makes it difficult to generalize findings to the country or disabled population as a whole. Therefore, it remains unclear whether an unmet need for family planning exists among women with disabilities, or how the various barriers discussed might perpetuate this inaccessibility (4). Despite having a national policy on disability and the rights of persons with disabilities incorporated in their national legal framework, Uganda still sees a lack of practice and application to achieve equity for women with disabilities in sexual and reproductive health care (7). In 2006, the Ugandan Disability Act was passed, enshrining in law equal access to a number of services Ugandans with disabilities, including healthcare. The act was further updated in 2019 to include additional provisions, but has been fraught with challenges regarding implementation and enforcement (8).

The aim of this study is to determine whether there is an association between disability severity and modern contraceptive use among women of reproductive age (15–49) in Uganda and the impact of this association on the odds of contraceptive use.

## 2 Materials and methods

This study analyzed the 2016 Uganda Demographic and Health Survey (UDHS) to examine disparities in modern contraceptive use based on disability severity. The 2016 DHS is the most recently completed full DHS survey conducted in Uganda, which is why this dataset was used. The UDHS is implemented by the Uganda Bureau of Statistics (UBOS) in collaboration with the Ministry of Health (MOH). The 2016 UDHS consisted of four questionnaires: the Household Questionnaire, the Woman's Questionnaire, the Men's Questionnaire, and the Biomarker Questionnaire. This study used data from the Household Questionnaire, which includes a set of questions developed by the Washington Group on Disability Statistics (WG) to determine the disability severity of all members in the sampled household, and the Women's Questionnaire, which collects data from all eligible women ages 15–49 in those households, including information on reproduction and family planning.

### 2.1 Sample

This study includes all women ages 15–49 years who completed the questions about contraceptive use and disability. Women who were pregnant at the time of the survey were removed from the sample as they would not be using contraception at the time of data collection. The final sample included 13,822 women.

### 2.2 Measures

#### 2.2.1 Outcome

The outcome assessed in this study is use of modern contraceptive methods. This is a bivariate outcome based on self-reported contraceptive use by the women in the survey. If women reported their primary contraceptive method as pills, an intrauterine device, an injection, female or male sterilization, an implant, female or male condoms, emergency contraceptives, or lactational amenorrhea, they were determined to be using a modern form of contraception. If they listed they used no contraception or any other method like periodic abstinence, withdrawal, or traditional contraceptive methods, they were determined to not be using a modern form of contraception. This study is focused on use of a modern contraceptive method for two reasons. First, reported use of non-modern methods was very low. Second, part of the purpose of this article is to determine use of contraceptive methods provided through the medical system, which would be limited to modern methods.

#### 2.2.2 Exposure

In 2014, DHS developed a disability module based on the Washington Group on Disability Statistics Short Set on Functioning (WG-SS). Uganda included this optional module in their 2016 DHS process. The disability module is used to determine whether or not a person has a disability, operationalized as a functional limitation, in six possible domains: seeing, hearing, mobility, cognition, communication, and self-care. The functional limitations are measured on a four-point scale: 0 (no difficulty); 1 (some difficulty); 2 (a lot of difficulty); or 3 (cannot do at all) (Washington Group documentation). The questions from the WG-SS were added to the DHS as a way to gather data regarding disability in a concise and easy to implement manner (9).

The Washington Group provides guidance for a number of ways data collected via the WG-SS can be analyzed. For this study, we elected to use a severity indicator, which places individuals into one of four categories of disability severity: none, milder, moderate, or more severe. Specifically, we used the Short Set Severity Category (SS-SC), which is based on the Short Set Severity Continuum (SS-SCo). The SS-SCo gives a score to each individual based on their responses regarding impact of any functional limitation in each of the domains. Those scores are then totaled, then placed into the categories of the SS-SC on the basis of cut points established by the Washington Group. The SS-SC was used because it is able to reflect the experiences of people with multiple disabilities more accurately by taking into account the collective impact of all potential functional limitations (10).

#### 2.2.3 Covariates

The following variables were included in the regression models: age (15–49 in five-year age groups); parity (no children, 1–3 children, or 4 or more children); residence (urban or rural); relationship status (never in a union, currently in a union, or formerly in a union); education level (no education, primary school, secondary school, or higher education); and wealth measured in quintiles.

### 2.3 Data analysis

Data from the 2016 Uganda DHS was accessed via the DHS website. The household and women's survey datasets were merged as the needed variables were in both datasets. Instructions provided by the DHS data team were used to create survey settings taking into account the clustering, stratification, and weights of the dataset (11). Analysis was conducted using Stata version 17.0.

We first analyzed descriptive statistics, including weighted percentages to take into account the survey design. Next, bivariate analysis was performed using chi-squares to determine if there was a significant association between modern contraceptive use, disability, and the other covariates. Crude and adjusted logistic regression models were created to calculate odds ratios. The crude model only included disability (measured with the SS-SC), while the adjusted model included disability and all of the covariates discussed above (age, parity, residence, relationship status, education, and wealth). These covariates were included in the adjusted models to control for the potential impact these other variables could have on use of contraceptives or on experiencing disability within the analysis sample.

## 3 Results

### 3.1 Demographics

Weighted percentages will be reported here, unweighted percentages can be seen in Table 1. Using the disability severity score measure, 20.51% of the women reported some type of disability, with 15.45% reporting a mild limitation, 4.52% a moderate limitation, and 0.55% a severe limitation. Almost three-quarters of the women lived in rural areas (72.83%). Over half of the women were currently in a union at the time of the survey (57.90%), while 27.86% were never in a union and 14.24% were formerly in a union. The average age of the women in the survey was 28 years. The average number of children the women reported was three, with 3,631 women reporting no children. Additional demographic information can be found in Table 1.

**Table 1:** Demographics.

Variable	Number	Percentage	Weighted percentage
Disability severity score			
None	10,786	78.04%	79.49%
Mild	2,280	16.5%	15.45%
Moderate	667	4.83%	4.52%
Severe	89	0.64%	0.55%
Total	13,822	100%	100%
Age			
15–19	3,294	23.83%	23.78%
20–24	2,655	19.21%	19.34%
25–29	2,183	15.79%	16.02%
30–34	1,869	13.52%	13.18%
35–39	1,526	11.04%	10.91%
40–44	1,322	9.56%	9.57%
45–49	973	7.04%	7.19%
Total	13,822	100%	100%
Parity			
No children	3,631	26.27%	26.51%
1–3 children	4,725	34.18%	34.78%
4+ children	5,466	39.55%	38.72%
Total	13,822	100%	100%
Residence			
Urban	3,357	24.29%	27.17%
Rural	10,465	75.71%	72.83%
Total	13,822	100%	100%
Relationship status			
Never in union	3,843	27.80%	27.86%
Currently in union	8,087	58.51%	57.90%
Formerly in union	1,892	13.69%	14.24%
Total	13,822	100%	100%
Highest education level			
No education	1,565	11.32%	9.80%
Primary	8,050	58.24%	56.82%
Secondary	3,196	23.12%	25.37%
Higher	1,011	7.31%	8.01%
Total	13,822	100%	100%
Wealth index			
Poorest	2,783	20.13%	16.81%
Poorer	2,694	19.49%	18.16%
Middle	2,542	18.39%	18.18%
Richer	2,632	19.04%	20.29%
Richest	3,171	22.94%	26.56%
Total	13,822	100%	100%

Approximately two-thirds of the women surveyed reported not using any form of contraceptive at the time of the survey. The most common method used was injections, with 2,082 (15.51%) women reporting this as their primary method. The next most common method as an implant, with 5.31% of women stating this was their primary method. Use of any modern method of contraception was reported by 4,097 women, or 30.43% of the sample. See Table 2 for more details regarding contraceptive methods.

**Table 2:** Contraceptive use.

Variable	Number	Percentage	Weighted percentage
Current contraceptive method			
Not using	9,295	67.25%	66.14%
Pill	215	1.56%	1.71%
IUD	154	1.11%	1.15%
Injection	2,082	15.06%	15.51%
Male condom	455	3.29%	3.53%
Female sterilization	296	2.14%	2.07%
Male sterilization	7	0.05%	0.06%
Periodic abstinence	144	1.04%	0.01%
Withdrawal	244	1.77%	2.13%
Other traditional	42	0.30%	0.29%
Implant/norplant	746	5.40%	5.31%
Lactational amenorrhea (LAM)	93	0.67%	0.70%
Female condom	1	0.01%	0.01%
Emergency contraception	8	0.06%	0.09%
Other modern method	1	0.01%	0.01%
Standard days method (SDM)	39	0.28%	0.29%
Total	13,822	100%	100%
Using a modern contraceptive			
No	9,725	70.36%	69.57%
Yes	4,097	29.64%	30.43%
Total	13,822	100%	100%

### 3.2 Bivariate analysis

Disability was not found to be significantly associated with modern contraceptive use. All of the covariates included in the model (age, parity, residence, relationship status, education, and wealth) were significantly associated with modern contraceptive use. Disability was only significantly associated with residence (*p* < 0.01) and wealth (*p* < 0.001). See Tables 3, 4 for these analyses.

**Table 3:** Bivariate Chi-square results—modern contraceptive Use and covariates.

Modern contraceptive use				
Variable	Using	Not using	Total	F-statistic
Disability				0.86
None	3,213 (79.65%)	7,573 (79.42%)	10,786 (79.49%)	
Mild	664 (15.08%)	1,616 (15.61%)	2,280 (15.45%)	
Moderate	198 (4.83%)	469 (4.38%)	667 (4.52%)	
Severe	22 (0.45%)	67 (0.59%)	89 (0.55%)	
Age				118.06***
15–19	322 (8.18%)	2,972 (30.60%)	3,294 (23.78%)	
20–24	885 (21.65%)	1,770 (18.33%)	2,655 (19.34%)	
25–29	903 (22.34%)	1,280 (13.26%)	2,183 (16.02%)	
30–34	760 (18.08%)	1,109 (11.04%)	1,869 (13.18%)	
35–39	574 (13.99%)	952 (9.57%)	1,526 (10.91%)	
40–44	462 (11.07%)	860 (8.91%)	1,322 (9.57%)	
45–49	191 (4.69%)	782 (8.29%)	973 (7.19%)	
Parity				378.85***
No children	279 (6.90%)	3,352 (35.08%)	3,631 (26.51%)	
3 or fewer children	1,741 (43.45%)	2,984 (30.98%)	4,725 (34.78%)	
4 or more children	2,077 (49.65%)	3,389 (33.93%)	5,466 (38.72%)	
Residence				11.60**
Urban	1,089 (30.02%)	2,268 (25.93%)	3,357 (27.17%)	
Rural	3,008 (69.98%)	7,457 (74.07%)	10,465 (72.83%)	
Relationship status				352.72***
Never in union	409 (10.03%)	3,434 (35.65%)	3,843 (27.86%)	
Currently in union	3,214 (77.62%)	4,873 (49.28%)	8,087 (57.90%)	
Formerly in union	474 (12.35%)	1,418 (15.07%)	1,892 (14.24%)	
Education				15.34***
No education	313 (7.27%)	1,242 (10.90%)	1,565 (9.80%)	
Primary	2,388 (55.89%)	5,662 (57.23%)	8,050 (56.89%)	
Secondary	1,024 (27.17%)	2,172 (24.56%)	3,196 (25.37%)	
Higher	372 (9.67%)	639 (7.28%)	1,011 (8.01%)	
Wealth				19.07***
Poorest	575 (11.94%)	2,208 (18.94%)	2,783 (16.81%)	
Poorer	774 (17.13%)	1,920 (18.61%)	2,694 (18.16%)	
Middle	791 (18.79%)	1,751 (17.91%)	2,542 (18.18%)	
Richer	896 (22.47%)	1,739 (19.34%)	2,632 (20.29%)	
Richest	1,061 (29.67%)	2,110 (25.20%)	3,171 (26.56%)	

*** *p* < 0.001; **p < 0.01; *p < 0.05.

**Table 4:** Bivariate Chi-square results—disability and covariates.

Disability—SSSC						
Variable	None	Mild	Moderate	Severe	Total	F-statistic
Age						0.75
15–19	2,567 (23.81%)	549 (23.93%)	149 (22.24%)	20 (26.91%)	3,294 (23.78%)	
20–24	2,078 (19.39%)	430 (19.05%)	129 (19.75%)	18 (16.87%)	2,655 (19.34%)	
25–29	1,722 (16.18%)	342 (15.52%)	106 (15.26%)	13 (14.02%)	2,183 (16.02%)	
30–34	1,442 (12.99%)	336 (14.48%)	82 (12.75%)	9 (8.19%)	1,869 (13.18%)	
35–39	1,187 (10.84%)	237 (10.32%)	89 (13.64%)	13 (15.28%)	1,526 (10.91%)	
40–44	1,015 (9.52%)	225 (9.55%)	70 (9.97%)	12 (13.73%)	1,322 (9.57%)	
45–49	766 (7.26%)	161 (7.15%)	42 (6.40%)	4 (5.00%)	973 (7.19%)	
Parity						0.62
No children	2,817 (26.33%)	622 (27.47%)	168 (25.81%)	24 (30.37%)	3,631 (26.51%)	
3 or fewer children	3,708 (35.00%)	768 (34.37%)	218 (33.10%)	31 (27.94%)	4,725 (34.78%)	
4 or more children	4,261 (38.67%)	890 (38.16%)	281 (41.09%)	34 (41.69%)	5,466 (38.72%)	
Residence						3.60*
Urban	2,699 (27.99%)	507 (24.13%)	136 (24.01%)	15 (20.17%)	3,357 (27.17%)	
Rural	8,087 (72.01%)	1,773 (75.87%)	531 (75.99%)	74 (79.83%)	10,465 (72.83%)	
Relationship status						0.44
Never in union	2,985 (27.74%)	655 (28.75%)	177 (26.33%)	26 (31.75%)	3,843 (27.86%)	
Currently in union	6,329 (57.90%)	1,310 (57.33%)	397 (59.87%)	51 (57.60%)	8,087 (57.90%)	
Formerly in union	1,472 (14.35%)	315 (13.92%)	93 (13.80%)	12 (10.64%)	1,892 (14.24%)	
Education						1.34
No education	1,244 (9.97%)	233 (9.14%	77 (9.28%)	11 (8.30%)	1,565 (9.80%)	
Primary	6,206 (56.07%)	1,386 (59.92%)	409 (58.98%)	49 (60.40%)	8,050 (56.82%)	
Secondary	2,552 (25.96%)	487 (22.90%)	137 (23.76%)	20 (23.00%)	3,196 (25.37%)	
Higher	784 (8.00%)	174 (8.05%)	44 (7.98%)	9 (8.30%)	1,011 (8.01%)	
Wealth						3.49***
Poorest	2,181 (16.53%)	455 (17.65%)	143 (18.79%)	14 (16.68%)	2,783 (16.81%)	
Poorer	2,051 (17.56%)	478 (20.29%)	143 (20.65%)	22 (24.77%)	2,694 (18.16%)	
Middle	1,928 (17.76%)	458 (19.90%)	137 (19.78%)	19 (17.98%)	2,542 (18.18%)	
Richer	2,044 (20.36%)	450 (20.76%)	120 (17.45%)	18 (20.20%)	2,632 (20.29%)	
Richest	2,582 (27.79%)	449 (21.39%)	124 (23.34%)	16 (20.37%)	3,171 (25.56%)	

*** *p* < 0.001; **p < 0.01; *p < 0.05.

### 3.3 Regression analysis

Women with disabilities did not have significantly different odds of using a modern form of contraceptive than women without disabilities in the crude model. This was true for women with mild (OR: 0.96; CI: 0.87–1.08), moderate (OR: 1.10; CI: 0.87–1.08), and severe (OR: 0.75; CI: 0.43–1.40) functional limitations. In the adjusted model, disability was still not significantly associated with use of modern contraceptives at the mild (OR: 1.00; CI: 0.89–1.13), moderate (OR: 1.12; CI: 0.92–1.38), and severe (OR: 0.77; CI: 0.41–1.43) scores. See Table 2 for more details regarding these models. See Table 5 for more details regarding the regression analysis.

**Table 5:** Logistic regression results.

	Crude	Adjusted
	*N* = 13,822	*N* = 13,822
F statistic	0.84	53.50***
Exposure		
Disability severity score (Comparison: None)		
Mild	0.96 [0.87–1.08]	1.00 [0.89–1.13]
Moderate	1.10 [0.92–1.32]	1.12 [0.92–1.38]
Severe	0.75 [0.43–1.30]	0.77 [0.41–1.43]
Covariates		
Age (Comparison: 25–29)		
15–19		0.70 [0.55–0.88]**
20–24		1.08 [0.92–1.25]
30–34		0.86 [0.74–1.01]
35–39		0.73 [0.61–0.88]**
40–44		0.63 [0.52–0.76]***
45–49		0.29 [0.23–0.36]***
Residence (Comparison: Urban)		
Rural		0.88 [0.76–1.02]
Wealth (Comparison: Middle)		
Poorest		0.54 [0.45–0.63]***
Poor		0.82 [0.71–0.94]**
Richer		1.12 [0.97–2.29]
Richest		1.08 [0.88–1.33]
Parity (Comparison: No Children)		
1–3 children		4.85 [3.83–6.13]***
4 + children		8.04 [6.12–10.57]***
Education (Comparison: No Education)		
Primary		1.71 [1.43–2.03]***
Secondary		2.15 [1.75–2.65]***
Higher		2.32 [1.80–3.00]***
Relationship (Comparison: Never in a Union)		
Currently in union/living with a man		1.68 [1.35–2.09]***
Formerly in union/living with a man		0.92 [0.72–1.16]

*** *p* < 0.001; **p < 0.01; *p < 0.05.

## 4 Discussion

Previous qualitative studies in Uganda have suggested disparities in access to reproductive health services on the basis of disability and that women with disabilities receive lower quality care. A study including persons with physical disabilities found limitations including transportation, costs, negative attitudes of providers, inaccessibility of buildings, and assumptions of asexuality (5). Humanity and Inclusion and ThinkPlace Kenya (12) had similar findings in their study including women with a variety of disabilities, adding communication barriers, social isolation, lack of education about reproductive health, and experience of gender-based violence as additional challenges. A 2014 study also found limited knowledge about pregnancy and contraceptives among women with disabilities, which would limit use of these services (13).

This study has found that there are no significant differences in use of modern contraceptives between women with and without disabilities. This is inconsistent with previous studies regarding this topic. A study in India found women with disabilities had lower odds of modern contraceptive use (3). Another study in the United States determined women with disabilities were less likely to receive family planning services than women without disabilities (14). These inconsistencies could be due to the different contexts and differential usage of contraception in the population as a whole in these environments. Also, there is variability in the method mix in these locations vs. Uganda. For example, female sterilization is the most common form of contraception used in India (15) while injections are the most common method in Uganda (4). The findings of this study are also contradictory to findings of another study in Uganda based on the 2011 DHS. This study only included women with disabilities, assessing prevalence of contraceptive use among women with disabilities and the factors impacting that use. This study found only 26.1% of their sample of women with disabilities reported contraceptive use, suggesting low uptake of these services. However, it did not compare women with and without disabilities to determine disparities in use of modern contraceptives, but instead focused on which variables had the greatest effect on contraceptive use (6). In a report analyzing disability and reproductive health using the DHS data in a number of countries, it was found that women with disabilities in Uganda had higher odds of contraceptive use (OR: 1.1, *p* = 0.014) (16).

While this study is not in line with the findings of other studies regarding contraceptive use and disability, there have been other studies that have not found differences in use of contraception between people with and without disabilities. For example, a study in Zambia focused specifically on condom use did not find statistically significant differences in use of condoms when comparing people with and without disabilities (17). This study included both women and men and used a binary disability variable, including those who stated they had “a lot of difficulty” or “cannot do at all” in any domain in the sample of people with disabilities in the study.

Other public health studies have operationalized disability differently than this study. For example, Ayiga and Kigozi (6) used data from the 2011 Ugandan DHS. This DHS also used the Washington Grop Short Set, but the authors of the study created a binary disability variable instead of using a severity score. Some studies that used the same data from the 2016 UDHS interpreted the disability variable differently. An analysis of use of HIV testing and counseling services using the 2016 Uganda DHS created a dichotomous disability variable, categorizing anyone who reported “some difficulty”, “a lot of difficulty” or “cannot do at all” in any domain as having a disability (18). The same strategy was used for a study evaluating household health financial risk and disability (19). While this is something the Washington Group states is possible, it is the most liberal way to define disability and is not highly recommended (20). Macquarrie and Fleuret (16) also used a binary disability variable in their study. Use of the severity indicator, while recommended by the Washington Group, is not as common as a binary disability variable. This study used the severity indicator as it does not oversimplify the experience of disability and can capture the unique experience of persons with chronic illness impacting multiple functions or multiple disabilities. It is possible that use of a binary disability variable could have identified greater differences in contraceptive use between women with and without disabilities depending on how liberally disability was defined. However, the authors believe it is important to capture the nuances of the impact of severity of disability on use of contraceptives as studies have shown the severity and visibility of a disability impacts the amount of discrimination experienced by people with disabilities in all aspects of their lives, including receiving quality healthcare (21).

### 4.1 Limitations

The DHS is a cross-sectional survey conducted approximately every five years with a new sample each time. Therefore, longitudinal analysis cannot be conducted and while associations can be assessed, causal relationships cannot be determined. While women in the survey identified themselves as having functional limitations, it is impossible to determine when this occurred and if it was true before or after they started using their current contraceptive method. It is also possible social norms around the definition of disability and stigma regarding disability impacted responses to the functional limitation question. As these questions are in the household section of the survey which is completed by one member of the household on behalf of the others, disability is being reported via a proxy (22). This is not best practice for these questions according to the Washington Group (20). Additionally, it may be difficult to compare previously existing studies to this study due to how disability was operationalized in terms of a severity indicator or binary disability variable, which may explain why data was inconsistent across studies. There also isn't data available regarding why women are or aren't using contraception or who made decisions regarding contraceptive use that could be included in this analysis. It could be that women with disabilities are using contraceptives at the same rate but for different reasons or that others are making this decision when compared to women without disabilities, but this could not be determined in this analysis.

### 4.2 Future research

Additional research should be conducted to determine if there are differences in contraceptive method mix, unmet need for contraceptives, or sustained use of contraception on the basis of disability status and disability severity. It is possible that even if women with disabilities use modern contraceptives at the same rates as women without disabilities, express needs for contraceptives at the same rate, or sustain use of their selected method in the long term as similar rates of as women without disabilities, there could be differences in types of contraceptives used by each group. In particular, there have been studies that suggest sterilization is a more commonly used method among persons with disabilities (23–25) and that this is not always a choice by the patient but rather their provider or family members (26–29). For example, a study in India found female sterilization use was more common among women with disabilities living in urban areas, where sterilization is less common in general (3). Female sterilization use was very rare in this dataset and therefore could not be analyzed as part of this study, but in other countries where sterilization is more common, research should be conducted on this topic to detect potential ethical or human rights issues.

### 4.3 Practice implications

While this study shows similar rates of contraceptive use among women with and women without disabilities, this does not mean that these groups of women have the same experiences with accessing reproductive health services like contraception. Negative attitudes and lack of education among health providers are consistently named as challenges persons with disabilities face when accessing reproductive health services, including contraceptives (2, 5, 30–32). It is important for reproductive health service providers, particularly those focused on provision of and education about contraceptives, be informed about the many challenges faced by women with disabilities when accessing these services. In particular, self-assessment for conscious or unconscious bias on the basis of disability status and the impact of the severity of disability is essential. Training on providing care to women with disabilities for providers as part of standard medical education would be a helpful part of this process. Particularly important are discussions of informed consent, consent vs. assent, and how to manage consent procedures for patients with disabilities (33). Obstetric providers have expressed a need for this type of training and motivation to participate in it in previous studies (34), implying it would be feasible to implement and providers would be open to pursue this training.

Regardless of comparison to women without disabilities, it is clear women with disabilities are using contraceptives. Therefore, it is essential for family planning programs and services to be accessible to persons with disabilities. For example, healthcare facilities should be designed to be accessible by all. Sign language interpreters should be available at facilities. Educational materials should be provided in a number of formats (visual, audio, written) and reading levels. Accessible transportation options should be available. Providing contraceptive care in patient's homes or local communities would help to negate some accessibility issues. National family planning programs should be assessed to identify potential barriers so they can be addressed.

## 5 Conclusion

Despite finding no significant difference in use of modern contraceptives on the basis of disability severity, there is still much we can learn from this study. Additional research should be conducted to determine if disparities exist in type of contraceptive used, sustainability of method, and unmet need for contraceptives. Studies should also be conducted in a variety of settings to identify international trends. Consistent use of disability measures in reproductive health surveys is essential to increase access to data for these studies. Healthcare providers need to receive training regarding working with patients with disabilities. Family planning programs and services should be accessible to persons with disabilities. Continued work in this arena is essential to ensuring all persons have access to the reproductive healthcare to which they are entitled.

## Data Availability

Publicly available datasets were analyzed in this study. This data can be found here: https://dhsprogram.com/data/.
